# Hyperkalemia in Heart Failure with Reduced Ejection Fraction Patients Treated with Sacubitril/Valsartan: Experience from a Tertiary Cardiac Center in Riyadh, Saudi Arabia

**DOI:** 10.3390/clinpract15100175

**Published:** 2025-09-24

**Authors:** Sarah M. Alyousif, Naif K. Alaqil, Mohamad Abdelshafy, Turki Alasmari, Naif H. Alqadhy, Nawaf S. Alzahrani, Mohammed A. Alhefdhi, Nawaf A. Alqahtani, Aamir Omair, Ahmed Alsaileek

**Affiliations:** 1 King Abdulaziz Cardiac Center, King Abdulaziz Medical City, Ministry of National Guard Health Affairs, Riyadh 14611, Saudi Arabia; 2 King Abdullah International Medical Research Center, Riyadh 11481, Saudi Arabia; 3 College of Medicine, King Saud bin Abdulaziz University for Health Sciences, Riyadh 11481, Saudi Arabia

**Keywords:** sacubitril/valsartan, heart failure with reduced ejection fraction, hyperkalemia

## Abstract

**Background/Objectives**: Heart failure with reduced ejection fraction (HFrEF) remains a major global health burden. Sacubitril/valsartan, an angiotensin receptor neprilysin inhibitor (ARNI), improves outcomes in HFrEF but may cause hyperkalemia. **Methods**: A single-center retrospective cohort study was conducted at King Abdulaziz Cardiac Center, Riyadh, including 238 HFrEF patients initiated on sacubitril/valsartan (2016–2021). Potassium levels were assessed pre-initiation and at 0–3, 3–6, and 6–12 months post-initiation. Hyperkalemia was analyzed at thresholds >5.0, >5.5, and >6.0 mmol/L. **Results**: Median age was 58 years (IQR 48–69); 75.2% were male. Hyperkalemia >5.0 mmol/L occurred in 44.4% (95% CI: 38.1–51.0) within three months post-initiation versus 8.2% (95% CI: 5.3–12.4) pre-initiation. Only 17.3% exceeded 5.5 mmol/L. Treatment discontinuation occurred in 7.1% of patients, with 1.3% stopping due to clinically significant hyperkalemia. McNemar’s test confirmed a significant increase in prevalence across time points (*p* < 0.0001). **Conclusions**: Despite increased hyperkalemia incidence, discontinuation rates were low, consistent with prior trials (PARADIGM-HF, PARAGON-HF). Sacubitril/valsartan remains an effective and generally safe therapy for HFrEF, with hyperkalemia manageable through monitoring. Limitations include missing potassium data, potential confounding factors, and the lack of a control group; future prospective studies with regular electrolyte monitoring are recommended.

## 1. Introduction

Heart failure can be defined as an abnormality of cardiac structure and/or function leading to failure of the heart to deliver oxygen at a rate commensurate with the requirements of the metabolizing tissues, despite normal filling pressures (or only at the expense of increased filling pressures). The epidemiology of heart failure in the Middle East is lacking, but it is estimated to be around 3.75 million patients [1]. One type of this syndrome is heart failure with reduced ejection fraction (HFrEF) characterized by ventricular dysfunction as a result of impairment in contractility [2]. The clinical features of HFrEF are dyspnea (orthopnea, paroxysmal nocturnal dyspnea) and fatigue [3]. Medical management of HfrEF has been proven effective and safe. However, there is an increased risk of drug–drug interaction causing adverse effects such as hyperkalemia. Hyperkalemia is defined when serum potassium ion (K^+^) levels are elevated more than the normal range (above 5.0 mmol/L). Several trials were conducted to assess the safety and efficacy of sacubitril/valsartan, a medication used to treat heart failure with reduced ejection fraction [4,5].

Sacubitril/valsartan is a medication categorized as angiotensin receptor neprilysin inhibitor (ARNI). The medication has been approved by the US Food and Drug Administration for HFrEF to lower exacerbation of symptoms and mortality. Sacubitril works by inhibiting neprilysin, limiting the breakdown of natriuretic peptides. As for valsartan, it acts by blocking Angiotensin II receptors [6]. Sacubitril/valsartan causes hyperkalemia mainly through ARB-mediated suppression of aldosterone, reducing renal potassium excretion. This effect is amplified in patients with impaired kidney function or on other potassium-retaining drugs.

Two trials have been conducted in 2014 and 2019; Paradigm [4] and Paragon [5] respectively. The Paradigm-HF trial concluded that sacubitril/valsartan yielded better results in preventing hospitalization (because of possible complications and side effects) and deaths in heart failure but a lower prevalence of hyperkalemia in contrast to enalapril, with a prevalence of 16.1% for potassium levels above 5.5 mmol/liter [4]. However, according to the Paragon-HF trial, sacubitril/valsartan did not lead to significantly lower hospitalizations and deaths, but it had a lower incidence of hyperkalemia in contrast to valsartan [5]. An analysis of the Paradigm trial concluded that Hyperkalemia in HFrEF is lessened by the use of neprilysin inhibitors when a combination of Mineralocorticoid Receptor Antagonists (MRA) with other RAAS (Renin–Angiotensin–Aldosterone) inhibitors are used [7]. A study conducted in King Fahad Cardiac Center, Riyadh, Saudi Arabia, which had results that were consistent with the Paradigm-HF trial, reported hyperkalemia, hypotension, and angioedema, with allergy and lesions being the most prevalent. However, the study outlined that the above side effects were non-serious [8]. A prospective study conducted in Saudi Arabia has reported a possible side effect of hypotension in one of the 61 patients, a majority of whom showed alleviation [9]. Extremely low and high levels of potassium are correlated with increased mortality rates [10]. A review article has concluded that, in terms of consistency across the various trials, sacubitril/valsartan has proven to be more effective in reducing hospitalization and mortality [11]. A multicenter retrospective cohort study has concluded that treatment with sacubitril/valsartan is associated with a significant lower rate of all-cause and heart failure admissions compared to angiotensin-converting enzyme inhibitors (ACEIs) and angiotensin receptor blockers (ARBs) [12].

An observational, retrospective, multi-center study based in Italy has found that, for HFrEF patients on sacubitril/valsartan, NYHA (New York Heart Association) classes improved within the first few months post-initiation, with left ventricular ejection fraction improving ≥5% in more than half of the total sample size. Additionally and more relevantly to the objective, 2.2% had hyperkalemia at characterization and 2.6% during follow up, confirming effectiveness and tolerability of the medication [13]. A prospective 6-month follow-up of 35 patients has concluded that sacubitril/valsartan was associated with improved left atrial and ventricular strain in patients with sinus rhythm [14]. A nationwide multi-center study was conducted in Japan to assess adverse events and drug continuation in heart failure patients on sacubitril/valsartan, which has shown that adverse events were reported in 22.5%, including hypotension, worsening renal function, hyperkalemia, and angioedema, with 22.6% of patients discontinuing with hypotension being the most common reason for discontinuation [15].

This study contributes to the growing body of evidence by identifying patients at higher risk of developing hyperkalemia after initiation of sacubitril/valsartan therapy and by examining its prevalence in a real-world cohort. The primary objective was to evaluate potassium levels in HFrEF patients following treatment initiation and to compare the prevalence of hyperkalemia before and after therapy in a larger sample from King Abdulaziz Cardiac Center in King Abdulaziz Medical City.

## 2. Materials and Methods

### 2.1. Study Design and Population

This study was a single-center study conducted in King Abdulaziz Cardiac Center, a tertiary cardiac center in King Abdulaziz Medical City in Riyadh, Saudi Arabia, which is considered one of the leading centers for contemporary cardiac care in the Middle East. in King Abdulaziz Cardiac Center started operating in 2001 and has a capacity of 124 beds. This was a retrospective cohort study aimed at assessing the prevalence of hyperkalemia in HFrEF patients treated with sacubitril/valsartan. The chosen period was from 2016 until the end of 2021. This study design was chosen to best accomplish the goal of this study, which was to observe and describe the pattern and prevalence of hyperkalemia over time in patients with HFrEF who were started on the medication using existing medical records to follow the same group of patients across predefined time intervals.

### 2.2. Patient Selection

A non-probability consecutive sampling technique was applied to include all HFrEF patients treated with sacubitril/valsartan, regardless of their age, gender, or nationality from 2016 until the end of 2021 in King Abdulaziz Cardiac Center. Pediatric patients were not included. All patients with HFrEF initiated on sacubitril/valsartan between 2016 and 2021 were screened. Patients were included in the analysis if they had at least one potassium measurement following drug initiation. A baseline potassium value was not required for inclusion; however, patients without baseline values were excluded from analyses requiring pre- and post- initiation comparisons. No imputation of missing laboratory values was performed. A total of 238 patients were included in the study. The data were collected in four time periods: pre-initiation of the medication (*n* = 233), within the first three months (*n* = 225), between three to six months (*n* = 171), and between six to twelve months (*n* = 176). Inclusion relied on having a potassium level in any of the four studied time periods.

### 2.3. Variables, Data Collection, and Ethical Considerations

The data were collected from the electronic medical records system after receiving approval from the Institutional Review Board of King Abdullah International Medical Research Center. Patients were given unique identifiers to ensure confidentiality, and consent was waived due to retrospective design of the study. The collected data included age at the time of initiation, gender, and BMI. The predefined time periods used to observe the potassium levels were four periods, starting at baseline pre-initiation, within the first three months of initiation, between three to six months, and finally between six to twelve months. Because potassium checks were clinician-driven rather than protocolized, the frequency and timing of testing varied among patients. This opportunistic testing may introduce bias, as patients perceived to be at higher risk could have undergone more frequent monitoring, potentially overestimating prevalence. Potassium values were extracted from the EMR at predefined time intervals (pre-initiation, 0–3, 3–6, and 6–12 months). No imputation was performed for missing laboratory values; analyses were conducted on a complete-case basis for each time window, with denominators reflecting the number of patients with available potassium measurements in that period. The comorbidities collected include diabetes mellitus, hypertension, chronic kidney disease, dyslipidemia, and hypothyroidism. The International Classification of Diseases, 10th Revision (ICD-10) was used for accurate diagnosis classification. The comorbidities were confirmed using ICD-10 codes and chart diagnosis. Other medications that the patients were actively taking alongside sacubitril/valsartan were collected and they include spironolactone, furosemide, insulin, beta-blockers, and metolazone.

### 2.4. Data Analysis and Presentation

Descriptive data analysis was conducted using Microsoft Excel (Microsoft Corp., Redmond, WA, USA) and inferential statistics were performed using IBM SPSS Statistics version 28. For categorical variables such as the status of hyperkalemia, medications, and comorbidities, they were presented in tables as counts and percentages. For ages and potassium levels, since most data were not normally distributed after testing for normality using the Shapiro–Wilk test, medians and interquartile range were used. Pie on pie chart was used to describe the percentage of medication discontinuation as well as reason of discontinuation. We compared within-patient change in hyperkalemia (>5.0 mmol/L; yes/no) from baseline to each window using McNemar’s test (paired nominal data; exact version when discordant counts were small). Tests were two-sided, α = 0.05. Prevalence is shown with 95% CIs. *p*-value < 0.05 was considered statistically significant. In addition to the primary analysis, a sensitivity analysis was performed including only patients with paired potassium values at baseline and within the first 3 months post-initiation. No imputation was applied; analyses were conducted on a complete-case basis.

## 3. Results

### 3.1. Patient Demographics and Characteristics at Baseline

A total of 238 patients were included in this study. Table 1 shows the median age of 58 years (IQR 48–69), while male sex predominated (75.2%). Body mass index had an average value of 30.0 ± 7.0 kg/m^2^. The comorbidities were diagnosed via ICD coding as a requested data set, and a minority of patients were clinically diagnosed. The most common comorbidities were diabetes mellitus, hypertension, and dyslipidemia, respectively. Moreover, a smaller proportion had other co-morbid conditions, such as chronic kidney disease and hypothyroidism as shown in Table 1. The majority of the patients were on beta-blockers (89.5%) and furosemide (89.5%), followed by spironolactone (77.7%). Insulin use was seen in 53.4% of the patients, while metolazone was used in 16% of the cohort. The most prevalent co-morbid conditions were diabetes mellitus (71.8%) and hypertension (70.6%). A total of 59.7% were documented to have dyslipidemia whereas 25.2% had CKD. Hypothyroidism was the least prevalent at 6.3%.

### 3.2. Potassium Levels and Prevalence of Hyperkalemia Pre- and Post-Initiation and Drug Discontinuation

Table 2 shows central tendency of serum potassium levels in the time periods studied as median and interquartile range. Discrepancies in patient numbers are due to retrospective nature of the study. At baseline, the median potassium level was 4.4 mmol/L (IQR, 4.1–4.7, range 3.0–5.7). Within the first 3 months, the median potassium level was 5.0 mmol/L (IQR 4.6–5.4, range 3.3–7.4). This shows an early rise in serum potassium levels post-initiation with sacubitril/valsartan.

Table 3 shows Patients who already had moderate hyperkalemia (K > 5.5 mmol/L) at baseline were only three patients (1.29%). The highest prevalence of mild hyperkalemia overall (K > 5 mmol/L) was reported within the first three months (*n* = 225) in 100 patients (44.4%). Out of the 225 patients who had an available serum potassium level within the first three months, 39 (17.3%) had a potassium level of more than 5.5 mmol/L and only 9 (4.0%) out of the 225 patients had severe hyperkalemia marked by a serum potassium level of more than 6 mmol/L within the first three months post-initiation.

**Figure 1:**
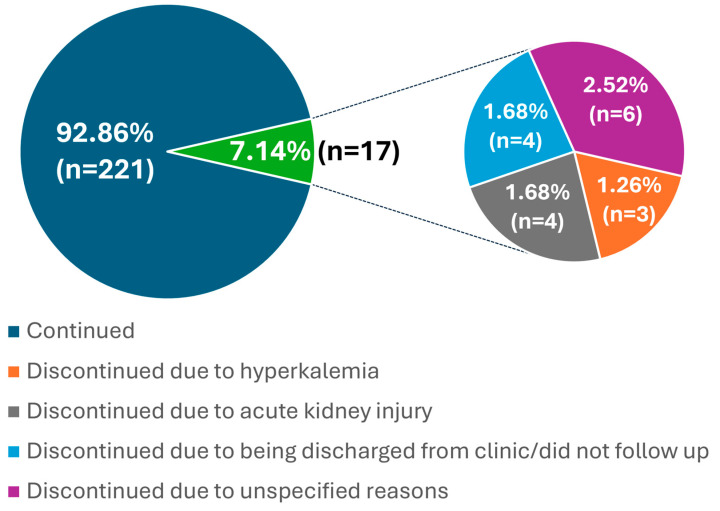
Proportion of Patients Continuing Medication vs. Discontinuing, and Distribution of Discontinuation Reasons *n* = 238.

Figure 1 shows that the proportion of patients who discontinued sacubitril/valsartan out of the total 238 patients were 7.1%, with 1.3% discontinuing due to clinically significant hyperkalemia, 1.7% due to acute kidney injury, 1.7% due to discharge/did not follow up in the clinic, and 2.5% due to unspecified reasons unrelated to safety.

### 3.3. Changes in Potassium Status

Table 4 illustrated the use of the McNemar’s test which showed a significant increase in hyperkalemic patients following initiation (*p*-value < 0.0001), with the highest incidence occurring within the first 3 months, while few patients with hyperkalemia at baseline eventually normalized potassium levels post-initiation. Within the first 3 months, 82 of 220 patients who were normokalemic at baseline developed hyperkalemia, while only 1 hyperkalemic patient normalized, resulting in a net increase of 81 cases. This further indicates the need for close potassium monitoring for patients on sacubitril/valsartan, especially within the first 3 months. Among 220 patients with both baseline and 0–3 month potassium values, hyperkalemia (>5.0 mmol/L) increased from 7.3% (95% CI: 4.5–11.5) at baseline to 44.1% (95% CI: 37.7–50.7) at follow-up. McNemar’s test confirmed a statistically significant increase (*p* < 0.0001). These results were consistent with the primary analysis, supporting the robustness of the observed association.

## 4. Discussion

This study aimed to assess the prevalence of hyperkalemia in patients with heart failure with reduced ejection fraction (HFrEF) treated with sacubitril/valsartan at King Abdulaziz Cardiac Center, a tertiary center in Riyadh, Saudi Arabia. The results show a statistically significant increase in patients becoming hyperkalemic following initiation of therapy, particularly within the first three months. Notably, 44.4% of patients developed hyperkalemia (K^+^ > 5.0 mmol/L) during that period, compared to only 8.15% before treatment initiation. Despite this increase, discontinuation rates remained low, with only 1.3% of patients discontinuing therapy due to hyperkalemia. The use of McNemar’s test provided evidence for the shift in potassium status post-initiation, highlighting the importance of close monitoring, especially in the first 3 months, to identify the patients who are at high risk of developing hyperkalemia and guide management plan. The sensitivity analysis restricted to patients with paired baseline and early follow-up potassium values confirmed the primary finding, demonstrating a substantial rise in hyperkalemia prevalence after sacubitril/valsartan initiation. By analyzing the same patients across both time points, this approach minimized potential bias from differing denominators due to missing laboratory data. The consistency between the sensitivity and main analyses strengthens confidence in the robustness of our results.

In terms of clinical implication, hyperkalemia clusters in the first three months after sacubitril/valsartan initiation, warranting a structured monitoring protocol. Serum potassium and creatinine should be obtained at baseline, at 1–2 weeks, at 4 weeks, and monthly through three months, with additional checks after dose escalations or intercurrent illness. Patients with chronic kidney disease or those receiving mineralocorticoid receptor antagonists and/or loop diuretics merit closer surveillance. Abnormal results should trigger medication review (including MRA titration and withdrawal of interacting agents), dietary counseling, and consideration of potassium binders where appropriate. Despite a higher frequency of early mild hyperkalemia, discontinuation was low, supporting continued sacubitril/valsartan therapy with proactive monitoring rather than routine cessation.

Compared with the randomized trials (PARADIGM-HF, PARAGON-HF) and real-world registries (REAL.IT, Italy; REVIEW-HF, Japan), our cohort shows a higher early prevalence of mild hyperkalemia after sacubitril/valsartan initiation (K^+^ > 5.0 mmol/L at 0–3 months: 44.4%). By contrast, moderate (>5.5: 17.3%) and severe (>6.0: 4.0%) events were less frequent, and discontinuation remained low (7.1% overall; 1.3% due to hyperkalemia), consistent with those reports. The most likely explanation is case mix: our patients had greater baseline MRA use (~78%) and more CKD (~26%), both of which elevate potassium risk. In addition, clinician-driven (non-protocolized) testing may have captured more early, mild elevations than trial settings with standardized monitoring. Importantly, a paired sensitivity analysis (baseline vs. 0–3 months, *n* = 220) showed the same signal (exact two-sided McNemar *p* < 0.0001), supporting robustness despite differences in testing strategy. As seen by prior literature, among hospitalized HFrEF patients, initiation of sacubitril/valsartan was associated with fewer admissions for heart failure, and was shown to be cost-effective in budget analysis, health care system, and societal perspectives [16].

In HFrEF cohorts, SGLT2 inhibitors show little direct effect on serum potassium in acute heart failure, while their natriuretic/diuretic profile may contribute to selective reductions in NT-proBNP—most evident in non-advanced disease [17,18]. By contrast, sacubitril/valsartan consistently lowers NT-proBNP (with BNP behavior influenced by neprilysin inhibition) and, in PARADIGM-HF, did not raise serum potassium overall, with treatment benefits preserved across the potassium spectrum [19,20]. These biomarker patterns help contextualize a higher early prevalence of mild hyperkalemia after initiation in routine practice: case-mix (CKD, MRA/diuretic exposure) and clinician-driven testing likely play a larger role than a potassium-raising effect of sacubitril/valsartan itself, while the low discontinuation rate supports continued therapy with monitoring [17,18,19,20].

Risk stratification is an important cornerstone in the management of HFrEF patients, as these patients are likely co-morbid with other conditions, especially conditions that predispose to hyperkalemia, such as chronic kidney disease and the use of spironolactone. Risk prediction models are suggested in the future to identify which patient is at risk of electrolyte disturbance post-initiation of the medication.

Certain strengths of the study include a large cohort (*n* = 238) for a single center study, with real-world data for high external validity. Our eligibility criteria allowed inclusion of patients with at least one post-initiation potassium value, even if baseline values were missing, in order to maximize sample size and better reflect real-world practice where laboratory monitoring may not always be complete.

As for the limitations of the study, this single-center, retrospective design limits causal inference and may introduce selection bias. Potassium monitoring was clinician-driven rather than protocolized, so testing frequency and timing varied; such opportunistic testing could overestimate early, mild hyperkalemia. Potassium values were missing at predefined windows; analyses used complete-case denominators with no imputation, and a paired sensitivity analysis (baseline vs. 0–3 months) yielded similar results, supporting robustness. Several potentially relevant factors were not captured, including dietary potassium intake, medication adherence, and the use or dose of potassium binders and over-the-counter supplements. Multivariable adjustment for case-mix (e.g., CKD, MRA use, concomitant therapies) was not performed, and no control group was available. Finally, hyperkalemia was not linked to clinical outcomes (e.g., emergency visits, hospitalizations, arrhythmias, or mortality), which constrains clinical interpretation of the biochemical findings.

The limitations of the study are summarized as the following:

1. The retrospective, single-center design limits causal inference and leaves room for selection bias.
2. Potassium monitoring was clinician-driven rather than protocolized, so variable timing and frequency may have preferentially captured early, mild hyperkalemia.
3. Potassium values were missing at predefined windows; complete-case analyses without imputation could shift prevalence estimates.
4. Important contributors—dietary potassium intake, medication adherence, and the use or dose of potassium binders or over-the-counter supplements—were not recorded, allowing residual confounding.
5. No multivariable adjustment or comparator cohort was included, limiting control for case-mix differences such as CKD, MRA exposure, and concomitant therapies.
6. Hyperkalemia was not linked to downstream clinical outcomes (e.g., emergency visits, hospitalizations, arrhythmias, or mortality), which constrains clinical interpretability.

This study contributes meaningful local data on the safety of sacubitril/valsartan in the local population. It shows the importance of risk stratification and monitoring in patients with HFrEF. These findings are in line with the practice of monitoring potassium levels post-initiation and suggest that further studies of prospective nature could help identify which patients are most at risk for complications and drug discontinuation.

Future research is suggested to be of prospective design to ensure regular follow-up, and with multi-center settings to include a wide range of demographics and investigate the possible role of demographic variability among different patient populations on outcomes, as well as to explore whether early hyperkalemia predicts long-term outcomes.

## 5. Conclusions

In this real-world HFrEF cohort, early mild hyperkalemia after sacubitril/valsartan initiation was common (K^+^ > 5.0 mmol/L: 44.4% at 0–3 months), whereas clinically significant elevations were less frequent (>5.5: 17.3%; >6.0: 4.0) and treatment discontinuation remained low (1.3% due to hyperkalemia; 7.1% overall). A paired sensitivity analysis confirmed the within-patient rise in hyperkalemia, supporting the robustness of these findings. Collectively with prior trials and registries, the results support continued sacubitril/valsartan use with structured potassium/creatinine monitoring during the first three months, especially in patients with CKD or receiving MRAs. Future prospective, multicenter studies linking biochemical changes to clinical outcomes are suggested.

## Figures and Tables

**Table 1:** Baseline Characteristics of Study Population (*n* = 238).

Characteristic	*n* = 238
Age at initiation in years	58 years (IQR 48–69)
Gender	
Male	179 (75.2%)
Female	59 (24.8%)
Body Mass Index (kg/m^2^)	30.0 ± 7.1
Comorbidities	
Diabetes Mellitus	171 (71.8%)
Hypertension	168 (70.6%)
Dyslipidemia	142 (59.7%)
Chronic Kidney Disease	60 (25.2%)
Hypothyroidism	15 (6.3%)
Medications	
Spironolactone	185 (77.7%)
Furosemide	213 (89.5%)
Insulin	127 (53.4%)
Beta-Blocker	213 (89.5%)
Metolazone	38 (16.0%)

**Table 2:** Serum Potassium Levels (mmol/L) at Baseline and Post-Initiation.

Time Period	Patients (*n*)	Central Tendency & Spread (Median, IQR)	Range (Min–Max)
Baseline	233	4.4 (4.1–4.7)	3.0–5.7
First 3 months	225	5.0 (4.6–5.4)	3.3–7.4
3 to 6 months	171	4.7 (4.4–5.1)	3.7–7.0
6 to 12 months	176	4.8 (4.5–5.2)	3.7–7.4

**Table 3:** Prevalence of hyperkalemia at predefined thresholds by time window.

Characteristic	Count (*n*), Prevalence (%)	95% CI (%)
Baseline Potassium (K) Levels (*n* = 233)		
K^+^ > 5 mmol/L	19 (8.2%)	5.3–12.4
K^+^ > 5.5 mmol/L	3 (1.3%)	0.4–3.7
Potassium Elevations Within First 3 Months (*n* = 225)		
K^+^ > 5 mmol/L	100 (44.4%)	38.1–51.0
K^+^ > 5.5 mmol/L	39 (17.3%)	12.9–22.8
K^+^ > 6 mmol/L	9 (4.0%)	2.1–7.4
Potassium Elevations Between 3 and 6 Months (*n* = 171)		
K^+^ > 5 mmol/L	46 (26.9%)	20.8–34.0
K^+^ > 5.5 mmol/L	16 (9.4%)	5.8–14.7
K^+^ > 6 mmol/L	6 (3.5%)	1.6–7.4
Potassium Elevations Between 6 and 12 Months (*n* = 176)		
K^+^ > 5 mmol/L	58 (33.0%)	26.4–40.2
K^+^ > 5.5 mmol/L	21 (11.9%)	7.9–17.6
K^+^ > 6 mmol/L	2 (1.1%)	0.3–4.0

Notes: CIs use the Wilson method. Denominators reflect complete cases per window (no imputation). K^+^: serum potassium.

**Table 4:** Paired change in hyperkalemia status (K^+^ > 5.0 mmol/L) vs. baseline after sacubitril/valsartan initiation.

Time Period	Patients with Paired Data	Normal → Hyperkalemia)	Hyperkalemia → Normal	Net Increase	*p*-Value
Within the First 3 Months	220	82	1	+81	<0.0001
Between 3 and 6 Months	168	38	10	+28	<0.0001
Between 6 and 12 Months	173	49	5	+44	<0.0001

Notes: Tests are exact, two-sided McNemar (α = 0.05). Denominators reflect complete cases per window, no imputation.

## Data Availability

The original data presented in the study are openly available in FigShare at https://doi.org/10.6084/m9.figshare.29920469.v1.

## Abbreviations

The following abbreviations are used in this manuscript:

HFrEF	Heart Failure with Reduced Ejection Fraction
ARNI	Angiotensin Receptor Neprilysin Inhibitor
RAAS	Renin–Angiotensin–Aldosterone
MRA	Mineralocorticoid Receptor Antagonist
ACEI	Angiotensin-Converting Enzyme Inhibitors
ARB	Angiotensin Receptor Blockers
ICD-10	International Classification of Diseases, 10th Revision
BMI	Body Mass Index
NYHA	New York Heart Association
